# Supplementary material for: Occludin Regulates HIV-1 Infection by Modulation of the Interferon Stimulated OAS Gene Family
Source: Mol Neurobiol. 2023 May 20;60(9):4966–82. doi: 10.1007/s12035-023-03381-0 (PMC10199280; doi:10.1007/s12035-023-03381-0)
Supplement: Supplementary file 1 — (PDF 83 kb) [file 12035_2023_3381_MOESM1_ESM.pdf]

Supplementary Table 1

Target gene	Assay ID	Reactivity
Ocln	Hs05465837_g1	Human
ZO-1	Hs01551867_m1	Human
OAS1	Hs00242943_m1	Human
OAS2	Hs00942643_m1	Human
OAS3	Hs00196324_m1	Human
OASL	Hs00984387_m1	Human
RNaseL	Hs00221692_m1	Human
IFNα5	Hs04186137_sH	Human
IFNα2	Hs00265051_s1	Human
IFNβ1	Hs01077958_s1	Human
STAT1	Hs01013996_m1	Human
STAT2	Hs01013116_g1	Human
